# The first complete mitochondrial genome of mushroom *Leucoagaricus naucinus* (Agaricaceae, Agaricales) and insights into its phylogeny

**DOI:** 10.1080/23802359.2021.1970643

**Published:** 2021-08-31

**Authors:** Cong Peng, Zhijie Bao, Wenying Tu, Lijiao Li, Qiang Li

**Affiliations:** School of Food and Biological Engineering, Chengdu University, Chengdu, Sichuan, PR China

**Keywords:** Agaricales, mitochondrial genome, phylogenetic analysis

## Abstract

*Leucoagaricus naucinus* (Fr.) Singer is a mycorrhizal fungus widely distributed in the northern Hemisphere. In the present study, the complete mitochondrial genome of *Leucoagaricus naucinus* was sequenced, assembled, and annotated. The *L. naucinus* mitochondrial genome was composed of circular DNA molecules, with the total size of 61,434 bp. The GC content of the *L. naucinus* mitochondrial genome was 26.07%. A total of 30 protein-coding genes (PCGs), two ribosomal RNA (rRNA) genes, and 26 transfer RNA (tRNA) genes were detected in the *L. naucinus* mitochondrial genome. Phylogenetic analysis based on combined mitochondrial gene dataset indicated that the *L. naucinus* exhibited a close relationship with *Agaricus bisporus*.

*Leucoagaricus naucinus* (Fr.) Singer is called Smooth Lepiota. This species is widely distributed in the northern Hemisphere, especially in China and North America (Vellinga et al. 2003), whose fruit bodies occur in autumn and can be found in spring and summer as well. Although this species is very common, the information of genetic characteristics of the species is very limited (Ning et al. 2016). It is recognized by its white cap, white gills, and white ring. The phenolic composition of the *L. naucinus* exhibited strong antimicrobial activity against some foodborne and spoilage bacteria (Aslim and Ozturk 2011). Up to now, the mitochondrial genome of *Leucoagaricus* genus has not been revealed. Mitochondrial genome has been widely used to analyze the phylogeny, evolution, and classification of fungal species (Zhang et al. 2017; Li et al. 2019b, 2020a). This study served as the first report on the complete mitochondrial genome from the genus *Leucoagaricus*, which will promote the understanding of phylogeny, evolution, and taxonomy of this important fungal species.

The specimen (*L. naucinus*) was collected from Sichuan, China (101.48 E; 27.41 N). The specimen was identified as *L. naucinus* by morphology, internal transcribed spacer (ITS) sequence and small subunit ribosomal RNA (rRNA) (*rns*) sequence. A specimen was deposited in collection center of Chengdu University (Qiang Li, leeq110@126.com) under the voucher number of s2148. We sequenced and *de novo* assembled the complete mitochondrial genome of *L. naucinus* according to previous described methods (Li et al. 2019a, 2019b; Wang et al. 2020a, 2020b). Briefly, the complete mitochondrial genome of *L. naucinus* was assembled using NOVOPlasty v4.3.1 (Dierckxsens et al. 2017; Li et al. 2020b), which was circularly assembled at the K-mer size of 27. We then annotated the protein-coding genes (PCGs), rRNA genes, tRNA genes, and introns of the *L. naucinus* mitochondrial genome using MITOS (Bernt et al. 2013) and MFannot (Valach et al. 2014), both based on the genetic code 4. PCGs or ORFs in the *L. naucinus* mitochondrial genome were also predicted based on the NCBI Open Reading Frame (ORF) Finder (NCBI Resource Coordinators 2018), and then annotated by BLASTP searches against the NCBI non-redundant protein sequence database (Bleasby and Wootton 1990). The tRNA genes in the *L. naucinus* mitochondrial genome were also predicted with tRNAscan-SE v1.3.1 (Lowe and Chan 2016).

The complete mitochondrial genome of *L. naucinus* is 61,434 bp in length. The base composition of the *L. naucinus* mitochondrial genome is as follows: A (37.82%), T (36.11%), G (13.56%), and C (12.51%). A total of 30 PCGs, two rRNA genes (*rns* and *rnl*), and 26 transfer RNA (tRNA) genes were detected in the *L. naucinus* mitochondrial genome. The complete mitochondrial genome of *L. naucinus* contained 18 introns, 14 of which were located in PCGs, including *cox1*, *cob*, and *nad5*. All of these introns were belonged to the group I, which were named according to previously described method (Zhang and Zhang 2019). To reveal the phylogenetic relationships of Agaricales species, we constructed a phylogenetic tree for 23 Agaricales species. *Hannaella oryzae* from the order *Tremellales* was set as outgroup (Li et al. 2021a). Bayesian analysis (BI) method was used to construct the phylogenetic tree based on the combined 14 core PCGs (Cheng et al. 2021; Li et al. 2021b, 2020c). Single mitochondrial genes were first aligned using MAFFT v7.037 (Katoh et al. 2019), and then concatenated into a gene dataset using the SequenceMatrix v1.7.8 (Vaidya et al. 2011). Best-fit models of evolution and partitioning schemes were detected using PartitionFinder 2.1.1 (Lanfear et al. 2017). We analyze the phylogenetic relationships of the 23 Agaricales species using MrBayes v3.2.6 (Ronquist et al. 2012) based on the combined gene dataset. As shown in the phylogenetic tree (Figure 1), the mitochondrial genome of *L. naucinus* exhibited a close relationship with *Agaricus bisporus*.

**Figure 1.**
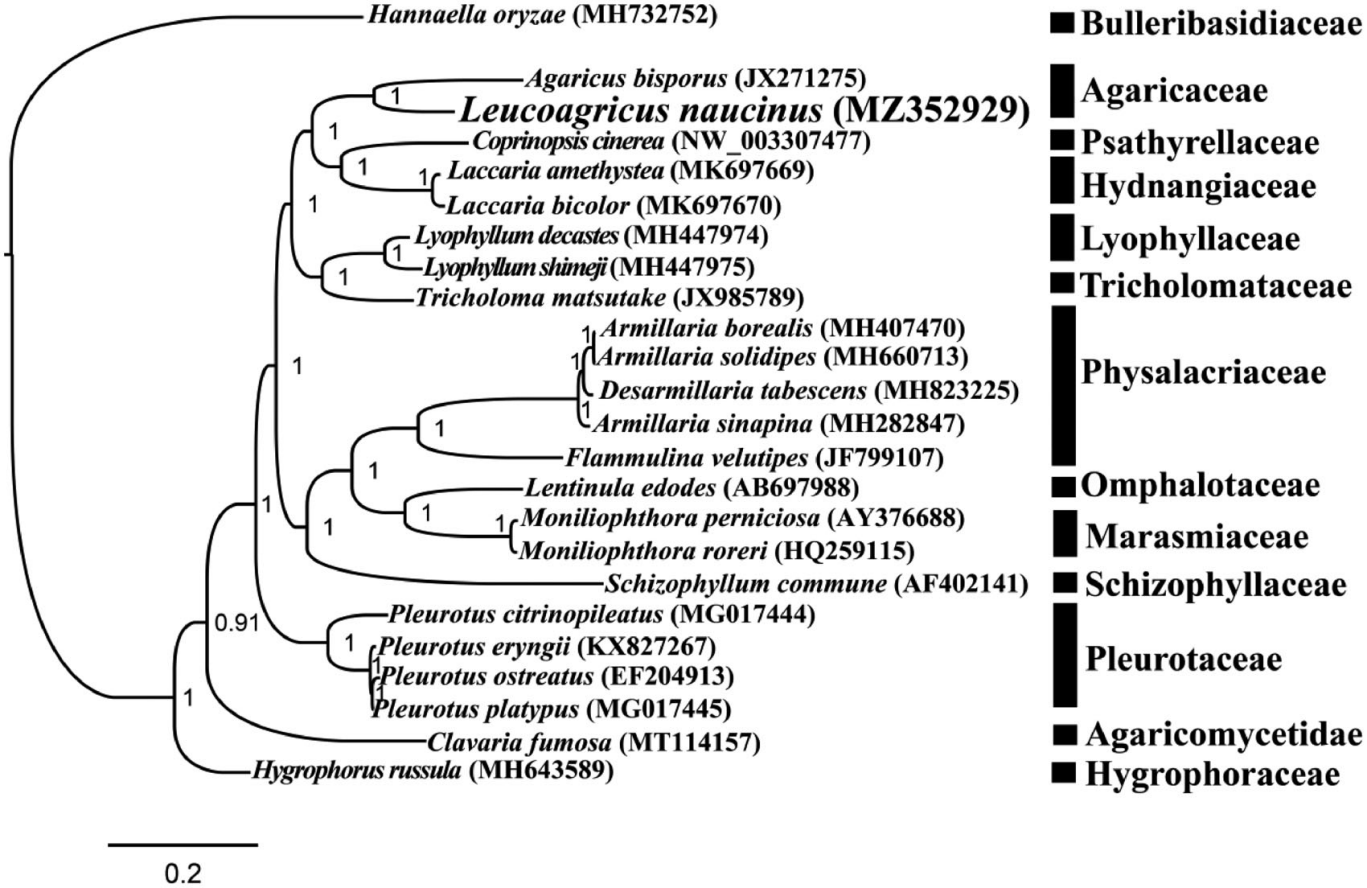
Bayesian phylogenetic analysis of 23 Agaricales species based on the combined 14 core protein-coding genes. Accession numbers of mitochondrial sequences used in the phylogenetic analysis are listed in brackets after species.

## Data Availability

The genome sequence data that support the findings of this study are openly available in GenBank of NCBI at https://www.ncbi.nlm.nih.gov/ under the accession no. MZ352929. The associated BioProject, SRA, and Bio-Sample numbers are PRJNA734961, SRR14737568, and SAMN19551202, respectively.
